# SAFE: SPARQL Federation over RDF Data Cubes with Access Control

**DOI:** 10.1186/s13326-017-0112-6

**Published:** 2017-02-01

**Authors:** Yasar Khan, Muhammad Saleem, Muntazir Mehdi, Aidan Hogan, Qaiser Mehmood, Dietrich Rebholz-Schuhmann, Ratnesh Sahay

**Affiliations:** 1 Insight Centre for Data Analytics, NUIG, Galway, Ireland; 2 AKSW, University of Leipzig, Leipzig, Germany; 3 Centre for Semantic Web Research, DCC, University of Chile, Santiago, Chile

**Keywords:** SPARQL query federation, Data access policy, Linked Data, Healthcare and life sciences

## Abstract

**Background:**

Several query federation engines have been proposed for accessing public Linked Open Data sources. However, in many domains, resources are sensitive and access to these resources is tightly controlled by stakeholders; consequently, privacy is a major concern when federating queries over such datasets. In the Healthcare and Life Sciences (HCLS) domain real-world datasets contain sensitive statistical information: strict ownership is granted to individuals working in hospitals, research labs, clinical trial organisers, *etc*. Therefore, the legal and ethical concerns on (i) preserving the anonymity of patients (or clinical subjects); and (ii) respecting data ownership through access control; are key challenges faced by the data analytics community working within the HCLS domain. Likewise statistical data play a key role in the domain, where the RDF Data Cube Vocabulary has been proposed as a standard format to enable the exchange of such data. However, to the best of our knowledge, no existing approach has looked to optimise federated queries over such statistical data.

**Results:**

We present SAFE: a query federation engine that enables policy-aware access to sensitive statistical datasets represented as RDF data cubes. SAFE is designed specifically to query statistical RDF data cubes in a distributed setting, where access control is coupled with source selection, user profiles and their access rights. SAFE proposes a join-aware source selection method that avoids wasteful requests to irrelevant and unauthorised data sources. In order to preserve anonymity and enforce stricter access control, SAFE’s indexing system does not hold any data instances—it stores only predicates and endpoints. The resulting data summary has a significantly lower index generation time and size compared to existing engines, which allows for faster updates when sources change.

**Conclusions:**

We validate the performance of the system with experiments over real-world datasets provided by three clinical organisations as well as legacy linked datasets. We show that SAFE enables granular graph-level access control over distributed clinical RDF data cubes and efficiently reduces the source selection and overall query execution time when compared with general-purpose SPARQL query federation engines in the targeted setting.

## Background

Inspired by the publication of hundreds of Linked Datasets on the Web, researchers have been investigating federated querying techniques to enable access to this decentralised content. Query federation aims to offer clients a single-point-of-access through which distributed data sources can be queried in unison. In the context of Linked Data, various optimised query federation engines have been proposed that can federate multiple SPARQL interfaces [1–7].

However, in the context of the Healthcare and Life Sciences (HCLS) domain – where data-integration is often likewise vital – the requirements for a federated query engine can be rather more specialised. First, real-world HCLS datasets contain sensitive information: strict ownership is granted to individuals working in hospitals, research labs, clinical trial organisers, *etc*. Therefore, the legal and ethical concerns on (i) preserving the anonymity of patients (or clinical subjects); and (ii) respecting data ownership through access control; are key challenges faced by the data analytics community working within the HCLS domain [8–11]. Second, many clinical datasets within the HCLS domain are composed of numerical observations that form multi-dimensional corpora of statistical information; for example, clinical trial data are often composed of various dimensions of patient attributes observed numerically along a temporal dimension. Thus to draw conclusions about biomarkers, side-effects of drugs, correlations within patient groups, *etc.*, requires applying statistical analyses to custom slices of multi-dimensional data.

The current research is then motivated by the needs of three clinical organisations: University Hospital Lausanne (CHUV)^1^, Cyprus Institute of Neurology and Genetics (CING)^2^, and ZEINCRO^3^. These organisations wish to develop a platform for analysing clinical data across multiple clinical sites, which would allow for increasing the total number of patients that are included in each analysis, thus increasing the statistical power of conclusions related to biomarkers, effectiveness and/or side-effects of drugs or combinations of drugs, correlations between patient groups, *etc*. The ultimate goal is to enable the collaborative identification of new drugs and treatments while reducing the high costs associated with clinical trials. With respect to this motivating scenario, the aforementioned general requirements apply: strict access control and adequate methods to represent and query statistical data across different sites are crucial aspects of this use-case.

Thus while query federation approaches enable the integration of data from multiple independent sources – as required by our motivating use-case – traditional federation approaches have not considered methods to enforce policy-based access control nor to deal *specifically* with statistical data – as also required by our use-case and in many other HCLS use-cases. Hence the central question tackled by this paper is how existing federated approaches can be modified to take both access control and statistical data into account while maintaining good performance characteristics.

The key challenges for federated querying (in generalised settings) are efficient source selection [7, 12] (i.e., determining which sources are (ir)relevant) and query planning (i.e., determining an efficient query execution strategy). These challenges change significantly when access control and statistical data are taken into account. More specifically, our hypothesis in this paper is that access control can facilitate more selective source selection by reducing the amount of data to be processed at an early stage of federated query processing, while statistical data represented as data cubes follow certain principles of locality that can be exploited to restrict the sources selected, reducing overall query time.

With respect to integrating policy-based access control, query federation engines often apply source selection at the level of endpoints, whereas in a controlled environment, a user may only have access to certain information *within* an endpoint. Adding an access control layer to existing SPARQL query federation engines thus adds unique challenges: (i) source selection should be granular enough to enable effective access control, and (ii) it should be policy-aware to avoid wasteful requests to unauthorised resources. However these challenges also present an opportunity to optimise the source selection process by increasing the granularity (in line with the access-control policies) and the selectivity (by quickly filtering resources to which users do not have access) of source selection.

On the other hand, with respect to statistical data, a common approach to representing such data is to use data cubes. In fact, the RDF Data Cube Vocabulary [13] has been standardised by the W3C precisely to enable the integration of multi-dimensional (e.g., statistical) data from multiple sites, as required by our HCLS use-case. Thus the statistical data from our use-case can be represented using this vocabulary, where existing federation engines could then be applied over the resulting sources (like any RDF dataset). However, our hypothesis is that the fixed structure of RDF data cubes implies certain locality conditions that are exploitable by the federated engine to optimise execution; in particular, we propose that specialised query planning for RDF Data Cubes can achieve better performance than a general federated query engine by taking such locality restrictions into account when selecting the sources to which individual triple patterns should be sent.

In this paper, we present *SAFE*: a *S*P*A*RQL Query *F*ederation *E*ngine that supports policy-based access to sensitive statistical data in a federated setting. As previously discussed, SAFE is motivated by the needs of three clinical organisations in the context of an EU project who wish to enable *controlled* federation over statistical clinical data – such as data from clinical trials – owned and hosted *in situ* by multiple clinical sites, represented in the form of data cubes. However, the methods proposed by SAFE can be used in other settings involving data cubes outside of the HCLS domain (even for open data).

Given that a wide variety of work has already been conducted on SPARQL query federation engines [1–7], it is important to highlight that our focus is on a higher level than such works: our core hypothesis does not relate to low-level join algorithms nor to communication protocols, for example, but rather focuses on the level of specialised source-selection and query planning algorithms for access-controlled federated over data cubes. Hence rather than propose and implement a federated query approach “from scratch”, we adapt a general-purpose query federation engine (FedX [1]), which already implements the low-level federation primitives that SAFE requires.

More specifically, SAFE extends upon the FedX engine [1] with two high-level novel contributions: (i) graph-level source selection, which is required to implement more granular access-control, and which is enabled by a novel data-summary generation technique and associated algorithm, (ii) optimisations for federated query processing over statistical data that are represented using the RDF Data Cube Vocabulary. With these modifications, we show that when compared with FedX, HiBISCuS [14] and SPLENDID [2], in such settings, SAFE can (i) support more granular graph-level access control than possible by simply layering access control on top of an existing engine that uses endpoint-level source selection, and can (ii) efficiently reduce the query execution time, the data summary generation time, and the overall data summary size, when federating specifically over RDF data cubes. We perform experiments with datasets and queries taken from our motivating use-case; to justify the claim that SAFE can be applied in other statistical scenarios, we additionally perform experiments over RDF data cubes taken from other domains.

In our initial work [15] SAFE has been evaluated against the FedX engine [1] which it extends. In this article, we extend our previous work by (i) improving the source selection algorithm and providing extended analysis thereof; (ii) developing an automated technique to generate data summaries (i.e., indexes) with lower relative sizes compared to the raw data and faster generation time; (iii) evaluating against two additional query federation engines (HiBISCuS and SPLENDID); (iv) increasing the number of queries and datasets for evaluation experiments. We highlight that contribution (ii) is particularly important when one considers updates to a source: while caching data summaries in the federated query engine has the advantage of enabling much more targeted source selection while minimising runtime queries to (potentially) remote sources, a disadvantage of using such summaries is the additional overhead of having to keep them up to date as the underlying sources change. This latter disadvantage can be partially mitigated by using lightweight summaries that are efficient to recompute over the updated sources.

The rest of the paper is structured as follows: “Motivating scenario” section discusses our motivational scenario where data from different clinical locations need to be queried and aggregated. “Related work” section discusses background and related work. “Methods” section presents the three main components of SAFE query planning. “Results” section presents evaluation of SAFE with respect to queries over various statistical datasets and “Conclusions” section concludes our work.

## Motivating scenario

As previously discussed, our work stems from the ambitions of three clinical organisations – University Hospital Lausanne (CHUV)^4^, Cyprus Institute of Neurology and Genetics (CING)^5^, and ZEINCRO^6^ – who are in the process of developing a platform for analysing clinical data across multiple clinical sites, allowing the reuse of remote data in a controlled manner. We now discuss important aspects of this use-case in the context of our research and in the context of the requirements it places on the SAFE federated engine.

### Use of Linked Data

With their stated goal of integrating clinical data in a controlled manner in mind, the three clinical organisations mentioned are partners in the Linked2Safety EU project^7^. The two main goals of the Linked2Safety project are (i) the discovery of data about eligible patients – also known as subject selection criteria – that can be recruited for clinical trials from multiple clinical sites; and (ii) enabling multi-centre epidemiological studies to facilitate better understanding of relationships between pathological processes, risk factors, adverse events, and between genotype and phenotype. Although Linked Data technologies can help enable multi-site interoperability and integration, the community largely focuses on datasets that can be made open to the public. In contrast, clinical data is often of an extremely sensitive nature and there is often strict legislation in place protecting the privacy of patients.

### Legal and ethical implications of patient privacy

According to EU Data Protection Directive 95/46/EC^8^, clinical studies that involve patient-specific information must adhere to data-access restrictions that preserve patient anonymity. More specifically, a data access mechanism must ensure that patient identity cannot be discovered by direct or indirect means using the dataset [16]. Similar legislation exists in other jurisdictions. To avoid sharing of individual patient records, the Linked2Safety consortium has developed a data mining approach for transforming raw clinical data into statistical summaries that may aggregate (or indeed redact) multiple dimensions of raw data as required for a particular application.

The result is a set of anonymised data cubes whose dimensions correspond to insensitive clinical parameters without personal information [17]. The resulting multidimensional output contains sufficient granularity to quickly decide if the dataset is relevant for a given analysis – e.g., to understand the scale and dimensions of the data – and to perform high-level meta-analysis of aggregated data. These data cubes are represented in a standard format – namely RDF Data Cube vocabulary per the recent W3C recommendation [13] – to enable interoperability (e.g., use of controlled vocabularies for dimensions) and to allow the later use of Linked Data publishing/access methods.

Although the data considered are aggregated and do not contain personal information about patients, deanonymisation may still be possible [18]: one cannot open a dataset and *fully* guarantee that it will not (indirectly) compromise patient anonymity [19]. Likewise, if a (bio)medical dataset necessarily involves genetic data, there exist identifying markers by which patients can be directly deanonymised; thus genetic data can only be pseudoanonymised [16]. Given such issues, in practice, sharing clinical datasets – even aggregated statistics – is often conducted under a strict legal framework between parties.

### Running example

In order to employ stricter data access restrictions on the anonymised multi-dimensional RDF data cubes, we require an access-control–based query-federation approach that enforces and optimises for restricted user access over these RDF data cubes. Likewise we wish to be able to optimise for certain locality conditions present in RDF representations of statistical data. To further illustrate and motivate, we now provide a walkthrough of an example that is representative of the main use-case scenario.

**Fig. 1:**
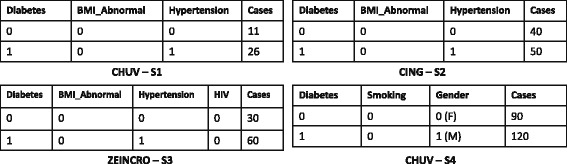
Example (2D) of data cubes published by CHUV, CING and ZEINCRO

Figure 1 shows four sample data cubes published by three different clinical sites. Each observation represents the total number of patients exhibiting a particular adverse event. For example, the CHUV-S1 observations describe the total number of patients (in the **Cases** column) that exhibit a particular combination of three adverse events: **Diabetes**, (Abnormal) **BMI_Abnormal** (Body Mass Index) and/or **Hypertension**. The value 0 or 1 indicates if the condition is present or not. For example, the second row in CHUV-S1 indicates that there are 26 cases presenting with both **Diabetes** and **Hypertension** but without **BMI_Abnormal**.

**Fig. 2:**
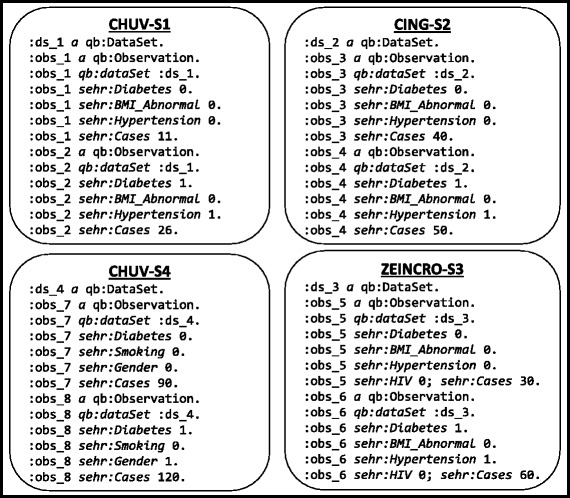
Datacubes represented using the RDF data cube vocabulary

These data cubes can be represented in the RDF Data Cube vocabulary [13], whose goal is to enable the interlinking and integration of statistical data cubes over the Web. The RDF resulting from representing the aforementioned data cube in this vocabulary is shown in Fig. 2.^9^ Individual data cubes are assigned to separate named graphs [20], where, e.g., :CHUV-S1, :CHUV-S4 are names for two RDF graphs from the same :CHUV source, whereas :CING-S2 is a another named graph published in a different source (:CING). Though not shown for brevity, each such graph is associated with its own provenance information. Each such named graph represents an independent data cube with disjoint sets of observations, encoded as RDF resources (e.g., :obs_7); this locality of data on information about observations suggests the possibility of optimising the source selection process to not only consider individual triple patterns, but rather observations as a whole when answering queries. However, it is important to note that the values of the dimensions (e.g., 1) and the properties used to capture the dimensions (e.g., sehr:Cases) are shared across data cubes, and thus across graphs and sources: locality does not apply in such cases.

As suggested by these example data, while the RDF Data Cube vocabulary provides terms to capture the structure of a data cube (which we discuss in more detail in “RDF data cubes” section), additional vocabulary is required to capture the clinical terminology needed by the clinical partners. Hence the Linked2Safety consortium has developed the *Semantic EHR Model* [21] (using the prefix “sehr” in Fig. 2) to capture a unified clinical terminology that covers the needs of the three clinical partners [21]; terms such as sehr:Cases, sehr:Diabetes, sehr:HIV, etc., constitute this vocabulary, capturing statistical dimensions of the clinical data cubes.

**Fig. 3:**
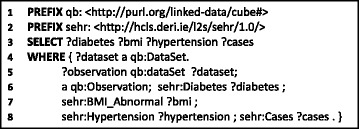
Example of subject selection criteria for clinical trials

Once the data cubes are published by clinical sites, query functionality must be made accessible to clinical researchers in a manner that can abstract the details of the underlying sources. Given that the underlying data are in RDF, a natural candidate is to use SPARQL [22]: the standard query language for RDF supported by a wide variety of tools. Figure 3 shows a sample SPARQL query specifying subject-selection criteria, asking for the counts of cases that involve some combination of diabetes, abnormal BMI, and hypertension. An answer returned by the query, i.e., number of cases, will play a major role in deciding the resources (i.e., number of subjects, location, etc.) required for conducting a clinical trial. However, answering such a query requires integrating RDF data cubes with three dimensions – **Diabetes**, **Hypertension**, **BMI_Abnormal** – and the respective counts originating from multiple clinical sites. For this, query federation techniques – that allow for answering queries over multiple independent data sources – will be required. Referring back to Fig. 1, only three of the data cubes (:CHUV-S1, :CING-S2 and :ZEINCRO-S3) contain all required dimensions. An answer returned by the query (Fig. 3) should list counts (i.e., *cases*) from these three RDF data cubes.

**Fig. 4:**
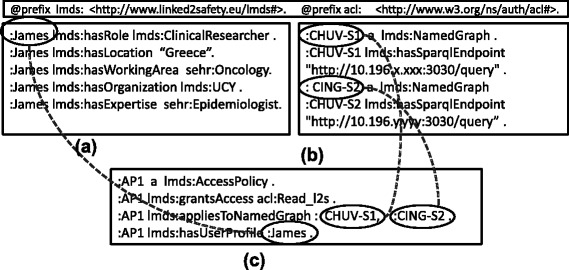
Snippets of user profile, access policy and data cube source information. **a** User profile, **b** Data cubes stored within named grap, **c** Access policy

However, as mentioned previously, these data cubes cannot be published openly on the Web, but are rather subject to strict access control policies. An important question then relates to how such a policy mechanism can be formulated over the RDF data cubes given in Fig. 2. Towards answering this question, the consortium has proposed the *Access Policy Model* (prefix “lmds”), which describes the users’ profiles (their activity, location, organisation, position and role) and their respective access rights (e.g., read, write) [23]. An example is provided in Fig. 4, where we see this model used to state that the user :James has read-level access to two named graph representing two independent data cubes residing in two locations: :CHUV-S1 and :CHUV-S2; by default, users do not have any privileges. In our scenario, the most common form of access-control policies are applied at the level of named graphs (i.e., data cubes).

Now when answering the SPARQL query in Fig. 3, we must take into account these data-access policies. For example, assuming the query is executed by the user :James, we know that he has access to the :CHUV-S1 and :CING-S2 RDF data cubes only. Therefore, the query federation engine should retrieve results only from :CHUV-S1 and :CING-S2 and should not consider :ZEINCRO-S3 for querying.

### Problem statement

In summary, from prior works we have a vocabulary to represent data cubes as RDF [13], a vocabulary to describe the clinical terminology of the partners in the project [21], a wide variety of proposals on how to execute SPARQL queries in federated settings [1–7], and a vocabulary for describing data-access policies over these data cubes [23]. However, we have no work looking at putting all these aspects together. Thus the motivation for our research is to investigate how to enable efficient access-controlled query federation over statistical data cubes. As argued in the introduction, we cannot use existing federation engines off-the-shelf since they do not provide source-selection with the granularity needed to implement graph-level access control. Likewise the regular structure of data cubes suggests optimisations that would not be possible in a more general RDF/SPARQL scenario. Hence the core research questions we tackle in this paper are:

- How can we efficiently implement source-selection in a federated scenario on the level of graphs (as needed to efficiently support graph-level access control)?
- Can we optimise the query federation process specifically for querying federated collections of RDF data cubes in a manner that allows us to outperform off-the-shelf engines?

Towards tackling these questions – questions that are key to realising the ambitions of the Linked2Safety project – we will later propose the SAFE query federation engine.

## Related work

The scenario described in the previous section touches upon three main areas: query federation, access control and data cubes. We now discuss related literature in these three areas, focusing on those works that deal in particular with the Semantic Web standards (e.g., with RDF and SPARQL) as relevant in our scenario.

### SPARQL query federation

Many query federation engines have been proposed for SPARQL (e.g., [1–7, 14, 24–27]). Such engines accept an input query, decompose it into sub-queries, decide relevance of individual data sources (typically considering sources at the level of endpoints) for sub-queries, forward the sub-queries to the individual endpoints accordingly and merge the final results for the query. Such engines aim to find and execute optimised query plans that minimise initial latency and total runtimes. This can be achieved by (i) using accurate source selection to minimise irrelevant messages, (ii) implementing efficient join algorithms, (iii) and using caching techniques to avoid repeated sub-queries.

Source selection is typically enabled using a local index/catalogue and/or probing sources with queries at runtime. The former approach assumes some knowledge of the content of the underlying endpoints and requires update/synchronisation strategies. However, the latter approach incurs a higher runtime cost, having to send endpoints queries to determine their relevance for various sub-queries. Thus, many engines support a hybrid of index and query-based source selection.

Table 1 gives an overview of existing SPARQL query federation engines with respect to source selection type, physical join operators, use of caching and explicit support for updates. We also remark on whether code is available for the system. In this setting, our work builds upon an existing federated engine – FedX – with support for an access-control layer over statistical data represented as RDF data cubes.

**Table 1:** Overview of existing SPARQL query federation engines

Systems	Source selection	Join type	Code	Policy	Cache	Update
ADERIS [27]	Index	Nested loop	✓	✗	✗	✗
ANAPSID [26]	Query & index	Adaptive	✓	✗	✗	✓
Avalanche [24]	Query & index	Distributed, merge	✗	✗	✓	✗
DARQ [4]	Index	Nested loop, bind	✓	✗	✗	✗
DAW [25]	Query & index	Based on underlying system	✗	✗	✓	✗
FedSearch [3]	Query & index	Bind, pull-based rank	✗	✗	✓	✗
FedX [1]	Query	Nested loop, bind	✓	✗	✓	✗
LHD [5]	Query & index	Hash, bind	✓	✗	✗	✗
SPLENDID [2]	Query & index	Hash, bind	✓	✗	✗	✗
HiBISCuS [14]	Query & index	Nested loop, bind	✓	✗	✓	✓
FEDRA [6]	Query & index	Nested loop, bind	✓	✗	✓	✓
SAFE [15]	Query & index	Nested loop, bind	✓	✓	✓	✓

### Access control for SPARQL

Various authors have explored access control models for SPARQL query engines. Gabillon and Letouzey [28] propose applying access control over named graphs and views, which are defined as graphs dynamically generated using SPARQL CONSTRUCT or DESCRIBE queries. Costabello et al. [29] propose SHI3LD: an access control framework for SPARQL 1.1 query engines that operates on the level of named graphs where permissions are based on the context of the user in the setting of a mobile device; permissions are checked using SPARQL ASK queries. Kirrane et al. [30] propose using stratified Datalog rules to enforce an access control model that operates over quad patterns, thus offering higher granularity of control. Bonatti et al. [31], propose “reactive policies” that can model, for example, access-control settings through an Event–Condition–Action paradigm. Daga et al. [32], on the other hand, propose to use the Datanode ontology – for describing data flows – to model policy propagation rules.

SAFE is designed specifically to query statistical RDF data cubes in a distributed setting, where access control is coupled with source selection and both operate on the same level of granularity: named graphs. Access control – deny or allow access – is based on user profiles and their access rights, which are described in the *Access Policy Model* created for the purposes of the Linked2Safety project [23].

### RDF data cubes

The RDF Data Cube Vocabulary (QB) [13] is a standard for describing data cubes as RDF, providing terms to represent the structure of such cubes in an agreed-upon manner, facilitating interoperability in the exchange and interlinkage of data cubes on the Web. We use qb: as a prefix to refer to this vocabulary. The core classes in the vocabulary are: qb:DataSet, whose instances represent individual data cubes; and qb:Observation, whose instances represent a coherent tuple of measurement values (as per the example in Fig. 2 where each observation refers to a tuple of values in the original data cubes of Fig. 1). In terms of describing individual observations – such as the type of measure (area, duration, volume, location, etc.), units of measure (m^2^, s, m^3^, lat/long, etc.), and so forth – QB recommends use of the Statistical Data and Metadata eXchange (SDMX)^10^ standard, which supports expressing such features in an interoperable manner. QB also allows for expressing further features of data cubes, with a prominent example being *slices*, where each slice is a group of observations with certain values on given dimensions that can, for example, be annotated with further meta-data or linked to/from other data; as a brief example, in Fig. 2, we could use QB to define a slice of of the cube :CING-S2 to represent statistics on patients with diabetes (with the value 1 for sehr:Diabetes), which would include :obs_4 but not :obs_3.

Although QB has been standardised relatively recently, there have been a few research works looking at exploiting data in this format. For example, a number of tools have been proposed to help users to create, publish and subsequently analyse RDF data cubes [33, 34]. On the other hand, Kämpgen et al. [35] look at methods to derive mappings to integrate disparate data cubes together into a global view. Relating more specifically to querying data cubes, Kämpgen and Harth [36] look at the performance of using traditional relational tools (MySQL/Mondrian) for OLAP-style queries with a platform using QB and an off-the-shelf SPARQL store (Virtuoso), including the effects of materialising views on query runtimes. In general however, while there have been some initial works looking to exploit RDF data cubes, to the best of our knowledge, no work has tackled the scenario of processing queries over a federation of data cubes that are access-restricted in various remote locations.

## Methods

**Fig. 5:**
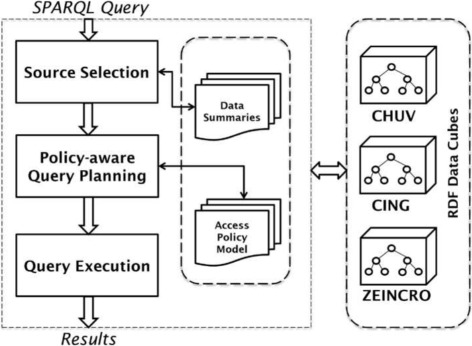
SAFE architecture

SAFE’s architecture is summarised in Fig. 5, which shows its three main components: (i) Source Selection: performs multilevel source selection based on the capabilities of data sources; (ii) Policy Aware Query Planning: filters the selected data sources based on access rights defined for each user; and (iii) Query Execution: performs the execution of sub-queries against the selected sources and merges the results returned. In the following, we describe these components in detail. The first two components (Source Selection and Policy Aware Query Planning) are described in Algorithm 2 whereas the third component (Query Execution) is delegated to the FedX query engine [1].

### Source selection

**Fig. 6:**
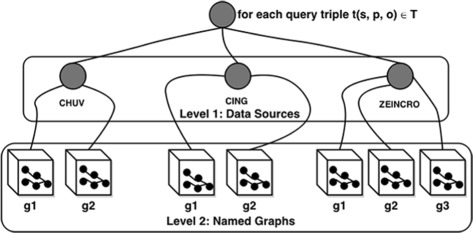
Tree-based two level source selection

SAFE performs a tree-based two-level source selection as shown in Fig. 6. At Level 1, like other query federation engines [1, 2, 5, 14, 26], we perform *triple-pattern-wise endpoint selection*, i.e., we identify the set of relevant endpoints that will return non-empty results for the individual triple patterns in a query. At Level 2 (unlike other query federation engines), SAFE performs *triple-pattern-wise named graph selection*, i.e., we identify a set of relevant named graphs containing RDF data cubes for all relevant endpoints already identified at Level 1. SAFE relies on data summaries to identify relevant named graphs.

#### Data summaries

SAFE’s data-summary generation algorithm is shown in Algorithm 1. The algorithm takes the set of all datasets (for example, CHUV, CING and ZEINCRO) as input and generates a concise data summary that enables graph-level source selection (as needed for the coupling with graph-level access control). By proposing a specialised algorithm for this setting, we claim that SAFE has significantly improved data-summary generation times when compared to other index-based approaches for general settings; this allows for faster recomputation of summaries when the underlying sources change. The data summaries themselves and the algorithm used to generate them are explained in the following.

We assume a set of datasets $\mathcal {D}$ where each dataset $D \in \mathcal {D}$ is a RDF dataset: *D*:={(*u*_1_,*G*_1_),…(*u*_*n*_,*G*_*n*_)}, where each (*u*_*i*_,*G*_*i*_) is a named graph with (unique) URI *u*_*i*_. In our case, named graphs refer to individual RDF data cubes as we do not consider a default graph. We denote all graph names by names(*D*), the set of all graphs by graphs(*D*), and a particular graph in the dataset by *D*(*u*):=*G*. We denote by preds(*G*):={*p*∣∃*s*,*o*:(*s*,*p*,*o*)∈*G*} the set of all predicates in *G* and, overloading notation, by $\text {preds}(D) := \bigcup _{(u,G)\in D}\text {preds}(G)$, we denote the set of all predicates in *D*. Finally, we assume that each dataset is published as an endpoint at a specific location, where loc(*D*) denotes the location (endpoint URL) of the dataset *D*.

For each dataset $D \in \mathcal {D}$, where each graph in *D* contains an RDF data cube, SAFE stores the following as a data summary: (i) the endpoint URL loc(*D*) (lmds:endpointUrl) (*line 4* of Algorithm 1); (ii) the set of all graph names (lmds:cube/lmds:graph) (*line 6* of Algorithm 1); and (iii) a map for each predicate appearing in the dataset to the set of names corresponding to the graphs in which it appears (lmds:cubeProperties) (*lines 7–8* of Algorithm 1).



**Fig. 7:**
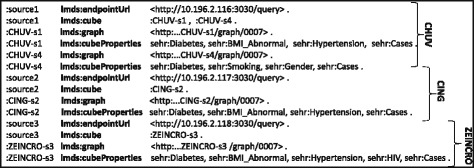
SAFE data summaries

We thus denote the set of all data summaries for $\mathcal {D}$ as $\mathcal {S}$ and the data summary for a particular source as $\mathcal {S}(D)$. A snippet of a data summary generated for the sample RDF data cubes published by three clinical sites (CHUV, CING, ZEINCRO) of Fig. 1 is shown in Fig. 7, where CHUV contains two RDF data cubes (:CHUV-S1, :CHUV-S4), CING contains one RDF data cube (:CING-S2), and ZEINCRO also contains only one RDF data cube (:ZEINCRO-S3).

From the collection of raw data summaries $\mathcal {S}$, we then compute some indexes that will be used to accelerate the source-selection process:

1. The set of all predicates in a given dataset *D*: preds(*D*). For instance, for the dataset CHUV from the running example (Fig. 1, Fig. 7), we have that preds(CHUV)={sehr:Diabetes, sehr:BMI_Abnormal, sehr:Hypertension, sehr:Smoking, sehr:Gender, sehr:Cases }.
2. The set of properties unique to a graph with name *u* in the dataset *D*, where, overloading notation: $\text {upreds}(u,D) := \{ p \in \text {preds}(D(u)) \mid \nexists u' : u' \neq u \wedge p \in \text {preds}(D(u')) \}$. For instance, from the running example, we have that upreds(:CHUV-S1,CHUV)={sehr:BMI_Abnormal,sehr:Hypertension} and upreds(:CHUV-s4,CHUV)={sehr:Smoking,sehr:Gender}.
3. The set of graph names in *D* that have at least one unique property: unames(*D*):={*u*∈names(*D*)∣upreds(*u*,*D*)≠*∅*}. For instance, from the running example, unames(CHUV)={:CHUV-S1,:CHUV-S4}.

These indexes will be used in the following source selection algorithm.

#### Source selection algorithm

SAFE’s source selection maps individual triple patterns to graphs within sources that contain data relevant for the query. However, in doing so, SAFE exploits certain locality properties exhibited by RDF data cubes: more specifically, we assume that subject–subject joins (henceforth: s–s joins) can only be satisfied by data local to a given RDF data cube. This locality assumption is based on the idea that data cubes stand as self-contained data structures within their respective named graph. More specifically, we assume that datasets, observations, slices, measures, etc., are not split over multiple data cubes/named graphs. For example, in Fig. 2, this assumption restricts the possibility of an observation instance, such as :obs_1, appearing as a subject in two distinct named graphs: in other words, the observation is assumed to be a local resource unique to a given data cube. This locality restriction applies equally within a dataset *D* and across all datasets $\mathcal {D}$.^11^ Hence when deciding the named graphs that may be relevant for a given triple pattern, we also consider other triple patterns that share the same subject; for example, in Fig. 3, triple patterns 2–7 (*lines 5–8*) form an s–s join and will be considered in unison, where although all sources will match the second and third triple pattern, these sources will not be considered relevant unless they are relevant for other triple patterns with the same subject.

The source selection process is detailed in Algorithm 1. The algorithm takes the set of all available datasets $\mathcal {D}$, their data summaries $\mathcal {S}$, and a a set of Basic Graph Patterns^12^ *BGP* as input. These BGPs correspond to all BGPs appearing in the input query, where each such BGP will be processed separately since it may correspond to, for example, optional or union patterns in the input query, rather than a standard join. The algorithm also accepts an access policy *P* and a user profile *U*; for the moment, we focus on the source selection, where we will provide more details of the user-level policies and access control in the section that follows. As output, the algorithm returns the set of relevant sources and corresponding named graphs identified for individual triple patterns.



We now discuss in detail the operation of the algorithm: *Line 1*: The source selection algorithm will return a set of candidate graphs for each triple pattern; these will be stored in $\mathcal {R}$, which is initialised on Line 1. The sources relevant for different BGPs will be kept separate in the results since different sources may be selected for the same triple pattern in two different BGPs. *Line 2*: The source selection algorithm will begin processing all BGPs in the query one-by-one. Each such BGP may refer to different parts of the query that may require a certain operation, such as a UNION or OPTIONAL clause, etc.; these will later be processed and combined by the FedX engine. *Lines 3–5*: Within each BGP, the algorithm proceeds by grouping triple patterns according to their subject and processing each subject-group separately. The algorithm first takes all triple patterns for a given subject and then extracts all (bound) predicates for that subject. *Lines 6–7*: For each dataset, if it contains all the predicate IRIs in the subject group, then it may contain relevant graphs (otherwise the algorithm continues to the next dataset). *Lines 8–18*: The algorithm uses information about graphs that contain unique predicates in a given dataset to potentially filter sources. If the subject-group contains such a predicate, then only that graph can be relevant. However, if the subject-group contains two (or more) predicates that are unique for different graphs, then no graph can contain relevant data and the algorithm proceeds to the next dataset. If no such predicates are found in the subject-group, potentially all graphs in the dataset are considered relevant. *Lines 19–24*: After all subject groups for the current BGP have been processed, for triple patterns with variables as predicates, some may be restricted to the sources of their subject group, while others may be matched to all possible graphs. In the latter case, to increase the selectivity of source selection, for such a triple pattern where at least the subject or object is bound, we will send an ASK query to each dataset to see if it may be relevant or not, *i.e.*, to see if the dataset contains data for that subject and/or object. If so, all graphs from that dataset are added. *Line 25*: The sources selected for the current BGP are added to the results. *Lines 26–28*: We will then check each graph selected (for some triple pattern in some BGP) against the policies and user profile, removing any graphs for which the user does not have access. We describe this process in more detail in “Security and authentication” section.

#### Source selection example

From our running example, let us consider the query shown in Fig. 3, which contains one BGP with seven triple patterns. The first step is to group the BGP into subject groups, which will result in two sub-BGPs, as follows:

**Table:** 

?dataset	a	qb:DataSet
?observation	qb:dataSet	?dataset
?observation	a	qb:Observation
?observation	sehr:Diabetes	?diabetes
?observation	sehr:BMI_Abnormal	?bmi
?observation	sehr:Hypertension	?hypertension
?observation	sehr:Cases	?cases

The first subject group will be matched to all graphs containing such a triple. For the second subject group, the set of all predicates is:



For each dataset, the algorithm checks to ensure that all predicates in the subject group are covered by the predicates in that dataset; this is the case for all three datasets in Fig. 2 (:CHUV, :CING, and :ZEINCRO). Again, given the locality restrictions, s–s joins are answerable only over a given dataset/graph, and hence we can safely filter other datasets from consideration for all triple patterns in that subject group.

Next, for each such dataset, the algorithm analyses graphs that uniquely contain predicates within that dataset. For example, for the :CHUV dataset, the first graph :CHUV-S1 contains the unique predicates sehr:BMI_Abnormal and sehr:Hypertension, while :CHUV-S4 contains the unique predicates sehr:Smoking and sehr:Gender. Since both sehr:BMI_Abnormal and sehr:Hypertension appear in the current subject group, only :CHUV-S1 will be selected as relevant for all triple patterns from the dataset :CHUV. This means, for example, that :CHUV-S4 will not be selected for the triple patterns referring to sehr:Diabetes, sehr:Cases, etc., even though data exists to match those patterns; due to the locality restrictions, such triple patterns cannot form a join with the sehr:BMI_Abnormal and sehr:Hypertension triple patterns.

Since no triple patterns contain unbound predicates, no ASK queries are sent. Then the selected sources are added for the current BGP. For the first triple pattern, all graphs in all datasets will be matched. For all triple patterns in the second subject-group, the following three dataset–graph pairs will be selected: (:CHUV,:CHUV-S1), (:CING,:CING-S2), (:ZEINCRO,:ZEINCRO-S3). Finally, user authorisation is checked for all graphs, where if authorisation is not available, the graph is filtered; we will discuss this access-control process in more detail in “Security and authentication” section.

Endpoints with selected named graphs will then be queried using standard federation techniques. For this, we use the FedX query engine [1], amending the query rewriter to append the relevant graph information for each endpoint.

#### Source selection correctness

The source selection algorithm assumes certain locality restrictions that must hold in the data for the algorithm to be correct. In particular, for a given set of datasets $\mathcal {D}$, we assume that if there exists a dataset *D*, a named graph name (*u*,*G*)∈*D*, and a triple (*s*,*p*,*o*)∈*G*, then there does not exist a dataset *D* ^′^, a named graph (*u* ^′^,*G* ^′^) and a triple (*s*,*p* ^′^,*o* ^′^)∈*G* ^′^ such that *D*≠*D* ^′^ or *u* ^′^≠*u*. In other words, we assume that subjects are unique to a given named graph (considering all named graphs and datasets in $\mathcal {D}$).

With this locality restriction, we can then begin to consider the correctness of Algorithm 2. The goal of the algorithm is to ensure that all possible answers for each BGP in the input (i.e., the answers possible for each BGP over a local merge of all data in $\mathcal {D}$) can be generated by joining the union of the results of individual triple patterns evaluated over the sources selected for those patterns. Once this process is assured, we delegate the processing of the query to FedX, which can use standard query execution methods to execute, for example, joins, left joins, unions, filters, etc., over the results of each BGP, producing the final query results for the user. First we must highlight that a known and non-trivial obstacle to completeness in federated scenarios is presented by blank nodes [37]; hence, per the running examples, we assume that no blank nodes are used in the data.

More formally, let $\mathfrak {D}$ denote the result of merging all datasets in $\mathcal {D}$ into a single global dataset^13^, let *b* *g* *p*={*t*_1_,…,*t*_*n*_} denote a BGP, and let $[\!\![{\mathfrak {D}}]\!\!]_{bgp}$ denote the result of evaluating *bgp* over $\mathfrak {D}$ [38]. Next let *R* denote the sources selected for *bgp* by Algorithm 2, let *R*(*t*) denote a dataset composed of the named graphs selected for the triple pattern *t* in *R*, and let [ [*R*(*t*)] ]_*t*_ denote the evaluation of triple pattern *t* over that dataset. The correctness condition we wish to satisfy is then as follows: $[\!\![{\mathfrak {D}}]\!\!]_{bgp} = [\!\![{R(t_{1})}]\!\!]_{t_{1}} \bowtie \ldots \bowtie [\!\![{R(t_{n})}]\!\!]_{t_{n}}$.

Let us start with a base case where the BGP has only singleton subject groups, meaning that no two triple patterns share a subject, and where all predicates are bound. In this case, the algorithm will select all datasets and (at least) all graphs with matching predicates. In this case, it is not difficult to show that $[\!\![{R(t)}]\!\!]_{t} = [\!\![{\mathfrak {D}}]\!\!]_{t}$ for all *t*∈*b* *g* *p*, and thus we have that $[\!\![{\mathfrak {D}}]\!\!]_{bgp} = [\!\![{\mathfrak {D}}]\!\!]_{t_{1}} \bowtie \ldots \bowtie [\!\![{\mathfrak {D}}]\!\!]_{t_{n}}$, which is the definition of the evaluation of BGPs [38]. Likewise, if we consider only singleton subject groups, but where some predicates are not bound, again the ASK queries will filter only irrelevant graphs, where it is again not difficult to show that $[\!\![{R(t)}]\!\!]_{t} = [\!\![{\mathfrak {D}}]\!\!]_{t}$ for all *t*∈*b* *g* *p* in this generalised case. In fact, these two base cases refer to standard techniques in federated SPARQL query processing.

What is left then is to verify the correctness of selecting sources by subject group. For the moment, we can assume that *bgp* forms one subject group and that all predicates are bound. Assume for the purposes of contradiction that as a result of Algorithm 2, $[\!\![{\mathfrak {D}}]\!\!]_{bgp} \neq [\!\![{R(t_{1})}]\!\!]_{t_{1}} \bowtie \ldots \bowtie [\!\![{R(t_{n})}]\!\!]_{t_{n}}$. First off, given that $[\!\![\!{R(t_{i})}]\!\!]_{t_{i}} \subseteq [\!\![\!{\mathfrak {D}}]\!\!]_{t_{i}}$ for 1≤*i*≤*n* (since *R*(*t*_*i*_) is a slice of data from $\mathfrak {D}$ and BGPs are monotonic), we have that $[\!\![{\mathfrak {D}}]\!\!]{bgp} \supseteq [\!\![{\mathfrak {D}}]\!\!]_{t_{1}} \bowtie \ldots \bowtie [\!\![{\mathfrak {D}}]\!\!]_{t_{n}}$, i.e., that we return only correct answers. Thus we are left with the case that $[\!\![{\mathfrak {D}}]\!\!]_{bgp} \subsetneq [\!\![{R(t_{1})}]\!\!]_{t_{1}} \bowtie \ldots \bowtie [\!\![{R(t_{n})}]\!\!]_{t_{n}}$. When could such a case occur? First of all, joins within a named graph between the triple patterns in *bgp* are not restricted by the algorithm, which only applies a trivial condition that all predicates in the subject-group be matched in the relevant datasets and that unique predicates in the graph are also satisfied. Hence for such a case to occur – for the algorithm to miss results – we would need a join to occur across graphs on (at least) the subject position. However, such a join would clearly break our locality restriction, and hence we have a contradiction. We highlight that it does not matter if the subject term is a variable or a constant here, nor does it matter if the constituent triple patterns in the subject group additionally have joins in other positions: such subject-groups can still only generate results with terms sourced from a single graph.

Finally, combining everything, we know that each sub-BGP pertaining to a subject group must return complete results, and hence the join of these sub-BGPs must also be complete. Thus the correctness condition (soundness and completeness) holds.

#### Source selection complexity analysis

With respect to the complexity of Algorithm 2, we analyse worst-case asymptotic runtime complexity considering RDF terms as symbols of constant length: *i.e.*, we do not consider the length of IRIs or literals since they are typically bounded by some small constant factor. To keep the analysis concise, we will consider *q* to be the size of the query encoding the number of triple patterns in the union of *BGP*; note that with this factor, we can abstract away the presence of multiple BGPs, the number of predicate in the query, etc., since these are bounded by *q*. Likewise we will consider *g* to be the total number of graphs in all datasets, and *d* to be the number of datasets available (note that *d* is bounded by *g*). Finally we denote by *p* the number of unique predicates in all graphs (more specifically, this is the cardinality of the set of all terms appearing in the predicate position of any graph) and by *t* the total number of triples in all graphs.

Creating the subject groups for a *BGP* and extracting the predicates for those groups can be done by sorting the triple patterns in the query, which is feasible in O (*q* log(*q*)) in the worst-case with, e.g., a Merge sort implementation.

For each subject group, we must check for each dataset that all predicates in that subject group are contained in the predicates for each dataset. This is feasible by a Merge sort over all predicates in the subject group (whose cardinality we denote by *p*_*s*_) with all predicates in the dataset, resulting in O ((*p*_*s*_+*p*) log(*p*_*s*_+*p*)) complexity for a given dataset and subject-group, or O (*d*((*p*_*s*_+*p*) log(*p*_*s*_+*p*))) for all datasets and a given subject group. When considering all subject groups, we can more coarsely (but concisely) represent the complexity as O (*q* *d*((*q*+*p*) log(*q*+*p*))), replacing both *p*_*s*_ and the number of subject groups with *q* by which both are bounded.

Next, for each graph with unique predicates, we need to check if any such predicate appears in the subject group. This is bounded by O (*q* *g*(*q*+*p*) log(*q*+*p*)) since we need to check each graph once (again, the first *q* bounds the number of subject groups, and the second and third *q* bound the number of predicates in each subject group).

Since the complexity O (*q* *g*(*q*+*p*) log(*q*+*p*)) asymptotically bounds the other factors, it represents the overall complexity up until Line 18 and thus the complexity of the analysis assuming all triple patterns have bound predicates and no access control is in place.

On Lines 19–24, the algorithm performs ASK queries to each dataset for each triple pattern matching the given criteria (bounded by *q*). In the general case, resolving ASK queries is NP-complete in combined complexity (considering both the size of the query and the data), even in the case that the query only contains a BGP and no features like optionals and filters; this is because each such query requires finding a homomorphism from the query graph to the data graph, where the graph homomorphism problem is NP-complete. However, since we only issue ASK queries with one triple pattern, and since the arity of the triple pattern is bounded, this step is feasible in (at least) time linear in the size of the data (for example, running a simple scan over all data), and so for all patterns, we have a resulting worst-case complexity of O(*qt*) from this part of the algorithm.

Hence before considering access control on Lines 26–28, we can merge these two factors to give a worst-case complexity of O (*q* *g*(*q*+*t*) log(*q*+*p*)). We emphasise that this is a coarse upper-bound in that it encapsulates disjoint cases whereby, e.g., a query’s subject groups repeat all predicates in the query and a query has no bound predicates, which is an impossible case to occur in one query; however, the complexity needs to bound all such cases since they affect different complexity parameters in different ways. In summary, as we will show, for real-world cases – where queries are small, predicates are few, and ASK queries are accelerated with pre-computed index schemes – the algorithm is much more efficient than this worse-case complexity bound may suggest.

Finally it is worth mentioning that the combined complexity for evaluating SPARQL queries is PSpace-complete [38], meaning that in a worst-case analysis, the actual evaluation of the query will dominate the source selection process. However again in practice, evaluating SPARQL queries can often be done efficiently in spite of such worst-case analyses: in particular, queries are often quite simple, having, for example, low *treewidth* (an indicator of how “cyclical” the interconnection of patterns in the query are), where queries with bounded treewidth are (often—depending on the exact query features permitted) *fixed-parameter tractable* [39]. The core conclusion is that worst-case analyses do not paint a complete picture: it is important to consider empirical performance results as well.

### Security and authentication

The proposed policy aware SPARQL federation functions on top of a security and authentication mechanism employed within the Linked2Safety software platform [40]. The security of user profiles, access policy, index (data summaries), and data cubes residing behind different SPARQL endpoints is based on a Public Key Infrastructure (PKI), which binds public keys with respective user identities by means of a Certificate Authority (CA). The user related information (profile and policy), data cubes, and data summaries are stored and hosted by the data owners, *i.e.*, by the healthcare organisation that gather the data. Each user identity must be unique within each CA domain maintained across organisations. For each user (profile), the user identity, the public key, their binding, validity conditions and other attributes are made un-forgeable through public key certificates issued by the CA. A log auditing mechanism keeps track of the query, the user and the data-cubes returned.

The process of allowing a user to access RDF data-cubes (and data summaries) is based on two axes: the first one is to authenticate the user, which allows the system to verify that the user is who he/she claims to be. This is also a prerequisite in order to know the role that an expert user has in the Linked2Safety system since after verifying the user, we can extract his/her role and corresponding data-access privileges. The second axis is to authorise the expert user to access the requested RDF data cubes if certain criteria based on his/her profile information are met, including role, working area, origin, etc. Each encrypted data-cube is sent (along with the signed hash-digest) with the public key of the client who requested the data (from his/her certificate). Upon successful verification, the expert user (profile) is authorised to perform a particular query over the SPARQL endpoint. The results are signed and encrypted by the clinical partners before being returned to the expert user, using the certificates. The Linked2Safety platform automatically verifies the origin of the data (non-repudiation), the sources they were sent from (authentication) and decrypts them (data integrity), before providing the query results.

**Fig. 8:**
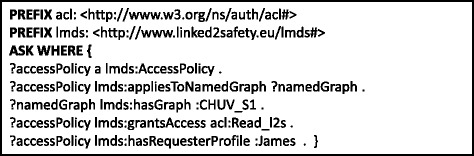
SPARQL query authenticating a user against a data cube/named graph

Again, the security and authentication platform for the Linked2Safety project is the subject of existing work [40], which SAFE considers as a black box. Integration with this platform is represented in Lines 26–28 of Algorithm 2, whereby applying source selection on the level of graphs allows the query federation process to be directly integrated with the graph-level access-control policies in place for the given stakeholders. An example of such access policies is given in Fig. 4 where we see that the user :James is permitted access only to two graphs: :CHUV-S1 and :CING-S2. In Fig. 8, we provide a SPARQL query that asks if the user :James has access to the graph :CHUV-S1; in the running example, this will return true. Let us consider again the example discussed for source selection where SAFE is executing the query in Fig. 3 over the federation of RDF data cubes outlined in Fig. 2. Without access control – prior to Line 26 in Algorithm 2 – the source selection algorithm will select the three dataset–graph pairs: (:CHUV,:CHUV-S1), (:CING,:CING-S2), (:ZEINCRO,:ZEINCRO-S3). However, the authenticated user does not have access to the latter source, and hence this graph will be filtered as a source, thus ensuring the user does not (attempt to) break the access policies of the stakeholders; if the source were not filtered, the request to access :ZEINCRO-S3 would rather be rejected at remote query-execution time.

Note that in the following evaluation section, we focus on the performance of the SAFE engine for executing federated queries and do not directly measure the performance of access control for the following two main reasons: (i) SAFE makes a sequence of calls to an external framework described in previous work [40] where such an evaluation would relate more to the external framework than to SAFE’s design; (ii) it is unclear to what we could compare the results since other federated query engines do not implement such access control. Instead, we highlight that the main contribution of SAFE for access control is implementing graph-level source selection, which enables tight integration with such an access control system; we will compare the performance of this more granular source selection in the following section with existing engines that offer dataset-level source selection.

## Results

In “Motivating scenario” section, based on our motivating scenario, we introduced two core research questions:

- How can we efficiently implement source-selection in a federated scenario on the level of graphs (as needed to efficiently support graph-level access control)?
- Can we optimise the query federation process specifically for querying federated collections of RDF data cubes in a manner that allows us to outperform off-the-shelf engines?

In terms of the first question, we have proposed the SAFE engine, which performs graph-level source selection that allows it to be integrated with graph-level access control; however, we have yet to see how efficient this alternative form of source selection when executing federated queries. In terms of the second question, we have proposed certain locality restrictions applicable in the context of RDF data cubes to refine the source selection process; however, we have yet to see how this optimisation compares to existing engines.

Along these lines, in this section we present the results of our evaluation comparing SAFE with three existing SPARQL query federation engines – FedX, HiBISCuS and SPLENDID – for a variety of queries and datasets along a series of metrics and aspects.

### Experimental setup

The experimental setup (i.e., datasets, setting, queries and metrics) for evaluation are described in this section. Note that the experimental material discussed in the following section and an implementation of SAFE are available online at https://github.com/yasarkhangithub/SAFE, which we will refer to in the following as the “homepage”.

**Datasets** We use two groups of datasets exploring two different use cases.

The first group of datasets (internal) are collected from the three clinical partners involved in our primary use case as described in “Motivating scenario” section. These datasets contain aggregated clinical data represented as RDF data cubes and are privately owned/restricted.

**Fig. 9:**
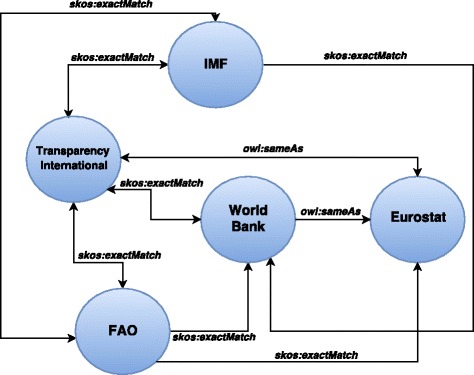
Linked Data cubes space

The second group of datasets (external) are collected from legacy Linked Data containing sociopolitical and economical statistics (in the form of RDF data cubes) from the World Bank, IMF (International Monetary Fund), Eurostat, FAO (Food and Agriculture Organization of the United Nations) and Transparency International. The World Bank data contains a comprehensive set of information about countries around the globe, such as observations on development indicators, financial statements, climate change, research projects, etc. The IMF data provides a range of time series data on lending, exchange rates and other economic and financial indicators. The Eurostat data provides statistical indicators that enable comparison between countries and regions across Europe. The Transparency International data includes a Corruption Perceptions Index (CPI), which ranks countries and territories based on how corrupt their public sector is perceived to be. The FAO data covers the areas of agriculture, forestry and fisheries. The Linked Data cubes space (Fig. 9) shows how these legacy datasets are interlinked with each other. These datasets provide links to each other using skos:exactMatch and owl:sameAs properties. The circles represent datasets while edges represent unidirectional or bidirectional links between any two datasets. These external datasets are available on the homepage.

Table 2 gives an overview of the experimental datasets, where we see that the largest dataset is IMF with 44 million triples describing 4 million observations with a total raw data size of 3.5 GB. On the other hand, the dataset with the highest dimensionality is FAO, with 247 unique properties. The largest INTERNAL dataset is CHUV with 0.8 million triples and 96 thousand observations; it also has the highest dimensionality, evidenced by 36 unique predicate terms.

**Table 2:** Overview of experimental datasets

Dataset	Type	№ trip	№ obsv	№ sub	№ pred	№ obj	Data		
CHUV	int	0.8 M	96 K	96 K	36	88	31 MB		
CING	int	0.1 M	17 K	17 K	21	51	5 MB		
ZEINCRO	int	0.4 M	49 K	49 K	24	59	15 MB		
Total	int	1.3 M	162 K	162 K	81	198	51 MB		
World Bank	ext	15 M	1.7 M	1.7 M	240	2.1 M	1.9 GB		
IMF	ext	44 M	4 M	4 M	181	1.4 M	3.5 GB		
Eurostat	ext	0.3 M	38 K	38 K	64	31 K	125 MB		
Trans. Int.	ext	43 K	3928	4198	49	11 K	121 MB		
FAO	ext	11 M	1.4 M	1.4 M	247	0.2 M	1.93 GB		
Total	ext	72 M	7 M	7 M	923	4 M	8 GB		

**Setting** Each dataset was loaded into a different SPARQL endpoint on separate physical machines. All experiments are carried out on a local network, so that network cost remains negligible. The machines used for experiments have a 2.60 GHz Core i7 processor, 8 GB of RAM and 500 GB hard disk running a 64-bit Windows 7 OS. Each dataset is hosted as a Virtuoso (Open Source v.7.2.4.2) SPARQL endpoint hosted physically on separate machines. Each instance of Virtuoso is configured with NumberOfBuffers = 680000, MaxDirty Buffers = 500000 and MaxQueryMem = 8G. Further parameters used to configure Virtuoso are available on the homepage.

**Queries** A total of 15 queries are designed to evaluate and compare the query federation performance of SAFE against FedX, HiBISCuS and SPLENDID based on the metrics defined. We define five queries for the federation of internal datasets (QL-*) and ten for the federation of external datasets (QE-*). The list of external queries is made available on the homepage. The queries are of varying complexity and have varying type of characteristics as noted in Table 3 where we summarise the characteristics of these queries following similar dimensions to that used in the Berlin SPARQL benchmark [41]. The row for the number of sources in this table indicates those matched by at least one triple pattern in the query.

**Table 3:** Summary of query characteristics

Characteristics	QE-1	QE-2	QE-3	QE-4	QE-5	QE-6	QE-7	QE-8	QE-9	QE-10	QL-1	QL-2	QL-3	QL-4	QL-5	
№ of Patterns	9	8	10	16	11	10	12	9	7	11	6	6	3	7	5	
№ of Sources	3	4	4	3	4	3	3	4	3	3	3	3	3	3	3	
№ of Results	41	1	10	41	64	22208	5	10	108779	4380	1701	17199	760	39312	10	
Filters		✓					✓			✓						
>9 pattens	✓		✓	✓	✓	✓	✓	✓		✓						
Negation							✓									
LIMIT modifier			✓				✓	✓							✓	
ORDER BY modifier			✓												✓	
DISTINCT modifier		✓		✓	✓	✓	✓	✓	✓		✓	✓	✓	✓	✓	
REGEX operator					✓					✓						
UNION operator	✓									✓						

**Metrics** For each query type we measured (i) the number of sources selected; (ii) the average source selection time; (iii) the average query execution time; and (iv) the number of ASK requests issued to sources. The performance of SAFE, FedX, HiBISCuS and SPLENDID was compared based on these metrics. The query results produced by SAFE, FedX, HiBISCuS and SPLENDID are the same for all queries.

### Experimental results

In this section, we present the experimental results gathered for the given datasets, setting, queries and metrics discussed previously.

**Index Generation Time and Compression Ratio:** SAFE’s index/data summaries generation approach is compared with various state-of-the-art approaches and the comparison results are shown in Table 4. The comparison metrics used are index generation time (**time**), index size (**size**) and the index reduction (**ratio**: computed as $1 - \frac {\text {index size}}{\text {total dump size}}$).

**Table 4:** Index generation time and compression ratio

System	Time	Size	Ratio
*External datasets*			
SAFE	102 s	23 KB	99.998%
HiBISCuS	1772 s	112 KB	99.994%
SPLENDID	369 s	252 KB	99.988%
FedX	–	–	–
*Internal datasets*			
SAFE	10 s	8 KB	99.984%
HiBISCuS	26 s	20 KB	99.961%
SPLENDID	74 s	21 KB	99.959%
FedX	–	–	–

As can be seen from the results, the index sizes for all approaches are much smaller than the relative size of the raw data dump. In the case of FedX, no indexes are created since relevant sources are determined on-the-fly at runtime. Aside from FedX, SAFE produces the smallest indexes by focusing only on meta-data about predicates and named graphs: for external datasets having a raw dump size of 8 GB, SAFE generates an index of size 23 KB, achieving 99.99% reduction, while for internal datasets, with a raw size of 51 MB, SAFE achieves a 99.98% (8 KB) index reduction. It should be noted however that although in relative terms HiBISCuS and SPLENDID produce indexes that are 2–11 times larger, the absolute sizes of the indexes are relatively small across all engines, where reduction rates remain above 99.9% for all three engines over all datasets.

As far as index generation time is concerned, aside from FedX which incurs no index generation costs, SAFE has a significant gain over all the approaches. In particular, with respect to the EXTERNAL datasets, SAFE’s index generation time is 102 s as compared to 1,772 s and 369 s for HiBISCuS and SPLENDID, respectively. SAFE has the lowest time for INTERNAL dataset as well though in overall terms, these times are quite small. Hence we see that, for example, upon updates to the INTERNAL federation of datasets, SAFE could recompute indexes from scratch in around 10 seconds. Of course for larger datasets, this (re-)indexing grows to the order of minutes.

While FedX incurs no index generation nor maintenance costs, we propose that SAFE’s indexes will reduce the load on remote endpoints and ultimately the overall query-execution time, and thus presents a good trade-off in a federated setting. These claims will be explored in the context of subsequent metrics (namely number of ASK queries, source selection time and query execution time).

**Triple pattern-wise sources selected:** Table 5 shows the total number of triple pattern-wise (TP) sources selected by SAFE, FedX, HiBISCuS and SPLENDID for all the queries. For the purposes of comparability, we count the number of datasets selected as relevant sources since only SAFE additionally selects relevant graphs. The last column in Table 5 shows the average number of TP sources selected by each approach across all queries.

**Table 5:** Sum of triple-pattern-wise sources selected for each query

System	QE-1	QE-2	QE-3	QE-4	QE-5	QE-6	QE-7	QE-8	QE-9	QE-10	QL-1	QL-2	QL-3	QL-4	QL-5	Avg
SAFE	7	11	13	16	14	12	14	12	10	11	6	6	3	9	5	10
FedX	8	18	20	24	24	15	19	19	11	22	14	16	7	15	13	16
HiBISCuS	9	6	10	16	12	5	12	9	3	11	14	16	7	15	13	10
SPLENDID	8	13	20	24	18	16	20	19	12	22	14	16	7	15	13	16

FedX performs source selection at the triple-pattern-level using ASK queries for each triple pattern to find out precisely which sources can answer an individual triple pattern. Thus, on the level of individual triple patterns, FedX selects all and only the actual contributing sources for those patterns. However, these sources might not be relevant after performing a join between two triple patterns, i.e., results from some sources might be excluded after join. HiBISCuS uses a hybrid source selection approach by using both ASK queries and data summaries to prune the number of relevant sources. SPLENDID uses VOID descriptions of sources to identify the relevant sources for each triple pattern of the query. SPLENDID also make use of ASK queries for source selection in cases where the query has bound variables that are not covered in the VOID descriptions.

The results show that on average HiBISCuS and SAFE have better source selection algorithms in terms of the average number of sources selected (10 and 10, respectively). The results in Table 5 show that FedX and SPLENDID overestimate the set of sources that contribute to the final query results. In the query execution times section, we will see that source overestimation leads to higher query execution times. Thus both HiBISCuS and SAFE outperform FedX and SPLENDID (in the average case) by not only considering sources relevant to a given triple-pattern, but also the other triple patterns in the query. For example, by using join-aware source selection designed for RDF data cubes, SAFE manages to filter further potential sources that do not contribute to the end results.

Although SAFE does not have a clear advantage over HiBISCuS in terms of the number of datasets selected as sources, SAFE does have an extra advantage over the other engines not illustrated by Table 5: the SAFE source selection algorithm prunes sources at the granularity of graphs, further restricting the data to be considered beyond datasets.

**Number of SPARQL ASK requests:** Table 6 shows the total number of SPARQL ASK requests used to perform source selection for each query. FedX is an index-free approach and performs runtime SPARQL ASK requests during source selection for each triple pattern in query: hence without any indexes, FedX must run many more such queries than the other engines that do support index information. HiBISCuS uses a hybrid approach that uses both runtime SPARQL ASK requests and pre-computed data summaries during source selection for each triple pattern in a query. SPLENDID uses VOID descriptions as well as ASK requests in case of bound variables in the query for which VOID does not offer relevant information. Hence, by considering indexes, both HiBISCuS and SPLENDID greatly reduce the number of ASK queries used during source selection. On the other hand, SAFE uses data summaries for source selection, reverting to SPARQL ASK requests only when there is an unbound predicate in a triple pattern *and* no locality restrictions are found to apply on the subject group to which that pattern belongs. None of our evaluation queries have such a triple pattern; hence there are no SPARQL ASK requests for SAFE.

**Table 6:** Number of SPARQL ASK requests used for source selection

System	QE-1	QE-2	QE-3	QE-4	QE-5	QE-6	QE-7	QE-8	QE-9	QE-10	QL-1	QL-2	QL-3	QL-4	QL-5	Avg
SAFE	0	0	0	0	0	0	0	0	0	0	0	0	0	0	0	0
FedX	54	48	60	96	72	60	72	54	48	66	18	18	9	21	15	47
HiBISCuS	12	12	6	24	12	18	12	12	12	12	0	0	0	0	0	9
SPLENDID	17	16	25	12	24	25	23	14	27	32	14	16	7	15	13	19

Though flexible in the generic case – particularly in the case of frequent updates to underlying sources – index-free approaches can incur a large cost in terms of SPARQL ASK requests used for source selection, which can in turn increase overall query execution time.

**Fig. 10:**
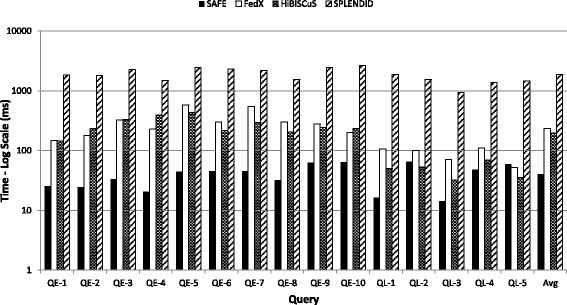
Comparison of source selection time

**Source selection time:** Figure 10 compares the source selection time for SAFE, FedX, HiBISCuS and SPLENDID for each query, where the *y*-axis is presented in log-scale. The rightmost set of bars compares the average source selection time over all queries. Given that the indexes of HiBISCuS, SPLENDID and SAFE remain quite small relative to total data sizes, they can easily be loaded into memory, where lookups can be performed in milliseconds. On the other hand, executing remote ASK queries are orders of magnitude more costly. Hence we see that the source selection time for SAFE is lower than the other approaches since SAFE uses ASK queries more sparingly, as previously discussed.

**Fig. 11:**
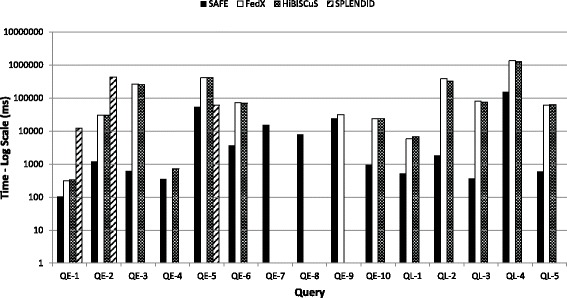
Comparison of query execution time

**Query execution time:** For each query, a mean query execution time was calculated for SAFE, FedX, HiBISCuS and SPLENDID by running each query ten times. Figure 11 then compares the mean query execution times of SAFE, FedX, HiBISCuS and SPLENDID for all queries. Again, the *y*-axis is log-scale. We set a timeout of 25 minutes on query execution; with these settings, FedX times-out in the case of four queries, HiBISCuS in three queries and SPLENDID in twelve queries (note that we do not show average execution times across all queries since it would be unclear what value to assign to queries that time-out). On the other hand, SAFE does not time out in any such case. Looking at query response times, SAFE outperforms the other engines in all queries. The fastest query (**QE-1**) is executed by SAFE within 100 ms, while the slowest query (**QL-4**) takes approximately 2 minutes. In total, SAFE executes 7 of the 15 queries in less than a second.

## Discussion

There are a number of factors that can influence the overall query execution time of a query federation engine, such as join type, join order selection, block and buffer size, etc. However, given that SAFE is based on the FedX architecture, we can attribute the observed runtime improvements to three main factors: (i) source selection time is reduced (as we have seen in the previous sets of results); (ii) fewer sources are queried meaning less time spent waiting for responses; (iii) source pruning at graph level within a source leads to querying fewer triples and (iv) triple patterns are more selective in SAFE, where, for example, our join-awareness makes it unlikely that all rdf:type triple patterns will need to be retrieved/queried for all sources but rather only from sources where such a triple pattern joins with a more selective one. Taken together, these four main observations explain the time saving observed for our presented use-cases, where the third and fourth observation in particular – the locality conditions on s–s joins designed for RDF data cubes combined with a more granular graph-level selection – play a significant role for restricting the amount of data generated for low-selectivity triple patterns. By making specific locality-based optimisations for the case of RDF data cubes, and combining this with finer-grained source selection on the level of graphs, SAFE can perform beyond what would is possible in off-the-shelf SPARQL federation engines designed for the general case.

Aside from query execution times, there are also a number of other important factors to consider, such as the load on the remove servers, and also the ability to cope with updates to the individual sources. In terms of load on the remove servers, we argue that by generating fewer ASK queries during the source-selection process, and in general by applying a more granular source-selection that requires processing fewer data, SAFE generates less load on remove servers, reducing the costs associated with hosting such services. With respect to updates, SAFE is less flexible than the index-free FedX approach, which requires no special action upon source updates; however, SAFE’s index is rather lightweight and can be recomputed from scratch (over an entire federation of sources) in the order of seconds for smaller datasets, and minutes for larger datasets, which should be acceptable except in the case that updates are very frequent and strong notions of distributed consistency are required. On the other hand, the benefit of SAFE’s indexing approach has been demonstrated in terms of source selection times, load on servers, query execution times, and so forth. Hence there is a clear trade-off, where we argue that in the case of SAFE, for most settings, the inflexibility with respect to updates is paid off in terms of overall efficiency.

Finally, the SAFE source selection algorithm has the additional advantage of allowing tight integration with a graph-level access-control framework. While this access-control requirement was the original motivation behind the design of SAFE, and in particular its graph-level granularity, as these experiments have shown, selecting sources on the level of graphs, in combination with join-aware optimisations, also gives general performance benefits even in the case that access-control is not needed.

## Conclusions

In this paper, we have presented SAFE: a query federation engine that enables policy-based access to sensitive statistical datasets represented as RDF data cubes. The work is motivated in particular by the needs of three clinical organisations who wish to develop a platform for collaboratively analysing clinical data that spans multiple clinical sites, thus improving the statistical power of conclusions that can be drawn (versus one source alone). Clinical data – even in aggregated form – is of a highly sensitive nature, and thus query federation engines must take access policies into account.

In our initial work [15] SAFE has been evaluated against the FedX engine, in this article, we extend our previous work by (i) evaluating against two additional query federation engines (HiBISCuS and SPLENDID); (ii) increasing the number of queries and datasets for evaluation experiments; (iii) presenting a variety of improvements and extended analyses for the data summary computation and source selection procedures.

SAFE is developed as an extension on top of the FedX federation engine to support two main features: (i) optimisations tailored for federating queries over RDF data cubes; and (ii) source selection using highly compressed data summaries on the level of named graphs that allows for integration with an existing access control layer. We evaluated these extensions based on our internal data sets (private data owned by clinical organisations) as well as external data sets (public data available from the LOD cloud) in order to measure the efficiency of SAFE against FedX, HiBISCuS and SPLENDID. Our evaluation results show that, for our use-case(s), SAFE outperforms FedX, HiBISCuS and SPLENDID in terms of source selection and query execution times.

In terms of future work, there are still a number of possible routes to explore with respect to improving the performance of SAFE. For example, in considering RDF data cubes described in individual named graphs, we currently only include locality restrictions on subject groups (s–s joins); however, there is the possibility to enforce such restrictions on other types of joins when they involve terms from the QB vocabulary, such as, for example, s–o joins on predicates like qb:dataSet. Furthermore, the structure of such data also suggests a closer look at the underlying join operator implementations: rather than relying on the general FedX query processor, SAFE could instead benefit from, for example, the ability to push joins to remove sources following available locality guarantees.

## Endnotes

^1^ http://www.chuv.ch/

^2^ http://www.cing.ac.cy/

^3^ http://www.zeincro.com/

^4^ http://www.chuv.ch/

^5^ http://www.cing.ac.cy/

^6^ http://www.zeincro.com/

^7^ http://www.linked2safety-project.eu/

^8^ http://www.dataprotection.ie/docs/EU-Directive-95-46-EC/89.htm

^9^ We omit definitions of prefixes for brevity since they are inessential to the discussion.

^10^ http://www.iso.org/iso/catalogue_detail.htm?csnumber=52500

^11^ Currently we assume this locality of s–s joins occurs in all cases since we deal purely with RDF data cubes; however, our algorithm could be trivially extended to drop or relax this locality principle in the presence of certain predicates or on instances of certain classes that may be described in multiple named graphs.

^12^ http://www.w3.org/TR/sparql11-query/%23BasicGraphPatterns

^13^ A possible corner-case occurs here if graphs with the same name appear in multiple datasets, but we will assume that such a case does not occur.
